# A Neuroelectrical Brain Imaging Study on the Perception of Figurative Paintings against Only their Color or Shape Contents

**DOI:** 10.3389/fnhum.2017.00378

**Published:** 2017-07-25

**Authors:** Anton G. Maglione, Ambra Brizi, Giovanni Vecchiato, Dario Rossi, Arianna Trettel, Enrica Modica, Fabio Babiloni

**Affiliations:** ^1^Department of Molecular Medicine, Sapienza Università di Roma Rome, Italy; ^2^Department of Human Physiology, Sapienza Università di Roma Rome, Italy; ^3^Department of Anatomy, Histology, Forensic Medicine and Orthopedics, Sapienza Università di Roma Rome, Italy; ^4^BrainSigns, Sapienza Università di Roma Rome, Italy

**Keywords:** neuroaesthetics, electroencephalography, hearth rate, galvanic skin response, isolated coherence

## Abstract

In this study, the cortical activity correlated with the perception and appreciation of different set of pictures was estimated by using neuroelectric brain activity and graph theory methodologies in a group of artistic educated persons. The pictures shown to the subjects consisted of original pictures of Titian's and a contemporary artist's paintings (Orig dataset) plus two sets of additional pictures. These additional datasets were obtained from the previous paintings by removing all but the colors or the shapes employed (Color and Style dataset, respectively). Results suggest that the verbal appreciation of Orig dataset when compared to Color and Style ones was mainly correlated to the neuroelectric indexes estimated during the first 10 s of observation of the pictures. Always in the first 10 s of observation: (1) Orig dataset induced more emotion and is perceived with more appreciation than the other two Color and Style datasets; (2) Style dataset is perceived with more attentional effort than the other investigated datasets. During the whole period of observation of 30 s: (1) emotion induced by Color and Style datasets increased across the time while that induced of the Orig dataset remain stable; (2) Color and Style dataset were perceived with more attentional effort than the Orig dataset. During the entire experience, there is evidence of a cortical flow of activity from the parietal and central areas toward the prefrontal and frontal areas during the observation of the images of all the datasets. This is coherent from the notion that active perception of the images with sustained cognitive attention in parietal and central areas caused the generation of the judgment about their aesthetic appreciation in frontal areas.

## Introduction

Often happens that during the observation of a piece of artwork, such as a painting or a sculpture, one can have the experience of what it is beauty. A recent discipline, called neuroaestethic, combines the neuropsychological research a with aesthetics by investigating the “perception, production, and response to art, as well as interactions with objects and scenes that evoke an intense feeling, often of pleasure” (Chatterjee, 2011). This recently developed field seeks, among other things, the neural correlates of aesthetic judgment and creativity. It is argued that visual aesthetics, namely the capacity of assigning different degrees of beauty to certain forms, colors, or movements, is a human trait acquired (Cela-Conde et al., 2004). In fact, it could be influenced by different psychological traits (Chamorro-Premuzi et al., 2009) and motivations (Chirumbolo et al., 2014) rendering the experience of beauty a defining characteristic of humankind (Leder, 2013).

Among the different models for the aesthetic appreciation that have been proposed in literature, Leder et al. (2004) have recently proposed a psychological model of aesthetic appreciation to describe the cognitive processes experienced when viewing an artwork. Such model consists of five different stages and two explicit outputs. The processing steps include the perceptual analysis, integration implicit memory, explicit classification, cognitive mastering, and evaluation. The explicit outputs are aesthetic judgments and aesthetic emotion. Cognitive processes involved in this model, translate the aesthetic appreciation into an aesthetic emotion. In this model, aesthetic judgment is the result of cognitive assessment and the aesthetic emotion is a byproduct of the processing steps. However, this model is mainly theoretical and does not explicitly provide an explanation of how the brain engages in aesthetic appreciation.

In order to link more precisely some cerebral areas to specific functions during the evaluation of artistic artifacts, different Authors have investigated mainly with the use of functional Magnetic Resonance Imaging (fMRI) the neural correlates of artistic perception (Ramachandran and Hirstein, 1999; Kawabata and Zeki, 2004; Chatterjee, 2011). In particular, significant activation of the emotional circuits of the brain, including the bilateral insula was observed during the evaluation of an artistic work (Ramachandran and Hirstein, 1999). In addition, if the artwork involved faces or figurative art, a specific area of the brain devoted to the recognition of faces (right fusiform gyrus) was also consistently activated. (Ramachandran and Hirstein, 1999). Other cerebral areas were shown to be correlated to the aesthetic perception, including motor, prefrontal, orbitofrontal, and anterior cingulate cortices (Kawabata and Zeki, 2004; Cupchik et al., 2009; Kirk et al., 2009). For instance, the prefrontal cortex is known for its roles in the perception of colored objects, decision-making, and memory. Recent studies have also linked it to the conscious aesthetic experience because it is activated during aesthetic tasks such as determining the appeal of visual stimuli (Cupchik et al., 2009).

Additionally, the medial orbito-frontal cortex (OFC) has been also found to respond aesthetics stimuli. In fact, the current evidence linking the OFC to attributed hedonistic values across gustatory, olfactory, and visual modalities, suggests that the OFC is a common center for the assessment of a stimulus's value (Kawabata and Zeki, 2004). The perception of aesthetics for these areas must be due to the activation of the brain's reward system with a certain intensity. Several other areas of the brain have shown to be slightly activated during some studies related to the art appreciation as the anterior cingulate cortex (ACC) previously known for its involvement in the feeling of romance, and the left parietal cortex, whose purpose would be to direct spatial attention (Cupchik et al., 2009). Different artistic styles may also be processed differently by the brain. In a study between filtered forms of abstract and figurative art, the bilateral occipital gyri, left cingulate sulcus, and bilateral fusiform gyrus showed increased activation with increased preference when viewing art (Kirk et al., 2009). In artwork, as representational paintings, where there are high levels of visual detail an activation in the bilateral occipital gyri may be caused by the large processing requirements placed on the visual system (Ramachandran and Hirstein, 1999). Several areas of the brain have been shown to respond particularly to figurative art perhaps due to the brain's ability to make object associations and other functions relating to attention and memory. This form of stimuli leads to increased activation in the left frontal lobe and bilaterally in the parietal and limbic lobes (Vartanian and Goel, 2004). The parietal lobe is involved in spatial cognition and visual imagery. This involvement can be find during aesthetic perception (Ramachandran and Hirstein, 1999). Until now, only few studies have been conducted to determine the functional neuroanatomical correlates of aesthetic experience in visual arts, though various behavioral studies have been concerned with the perception of artworks and style related aspects of processing (Locher, 2003; Belke et al., 2006; Lengger et al., 2007).

In this context, we would like to investigate two issues related to the timing of the perception and to the generation of the aesthetic judgment in humans. The first issue is related to what specific elements of the perception in the appreciation of figurative paintings are correlated with regional brain activity during the time duration of the observation. The second issue is related to the study of the temporal sequence of the aesthetic judgment formation. In fact, it is know that 10 s are sufficient to obtain an overview of the picture (Vartanian et al., 2005) while 30 s is the average length of time required for an aesthetic judgment when unlimited time is given, as in the case of real art museum visitors (Leder et al., 2004). Thus, it could be of interest to investigate the neuroelectrical correlates of brain activity related to these two duration (e.g., 10, 30 s) of the observation of the pictures.

In order to approach such issues, the state of the art of the neuroelectric methodologies (Babiloni et al., 2001; Cincotti et al., 2008) has been applied for the gathering and the analysis of the cerebral activity during the observation and the aesthetic judgment of different series of images. Such images are taken from the figurative works of the ancient Italian painters Titian (1490-1576) and a contemporary one, Luisa del Campana. In addition, from those images two other set of images are derived, containing only the information related to the color and the shape of the previous images. Successively, we gathered the cerebral electrical and emotional correlates of the observation of such three image datasets in a group of art educated persons. In particular, we would like to answer to the following experimental questions:

1. Which neural correlates (if any) of the pleasantness perception will correlate to the verbal report given by the subjects after the experience?
2. Are there differences in the neural correlates of the perception of the proposed images after 10 or 30 s of observation?
3. Are there stable patterns of cortical connectivity during the evaluation of the proposed pictures after 10 or 30 s of observation?

In the following sections, the methodologies employed and the main results obtained will be presented and discussed.

## Materials and methods

### Participants

A group of 16 healthy subjects (7 male and 9 female; average age 38.3 ± 6 y.o.) were involved in this experiment. All the subjects had a specific university education in art at a bachelor level. All subjects participated voluntarily to the study and each one of them gave written informed consent in accordance with the Declaration of Helsinki of 1975, as revised in 2000. The research project related to this study has received the approval of the proper ethical committee of the University of Rome Sapienza. Furthermore, the ethical committee of the IRCCS Fondazione Santa Lucia also approved the same research study. In fact, IRCCS Fondazione Santa Lucia is the institution on which the actual EEG recordings were made.

### Stimuli preparation

**Figure 1:**
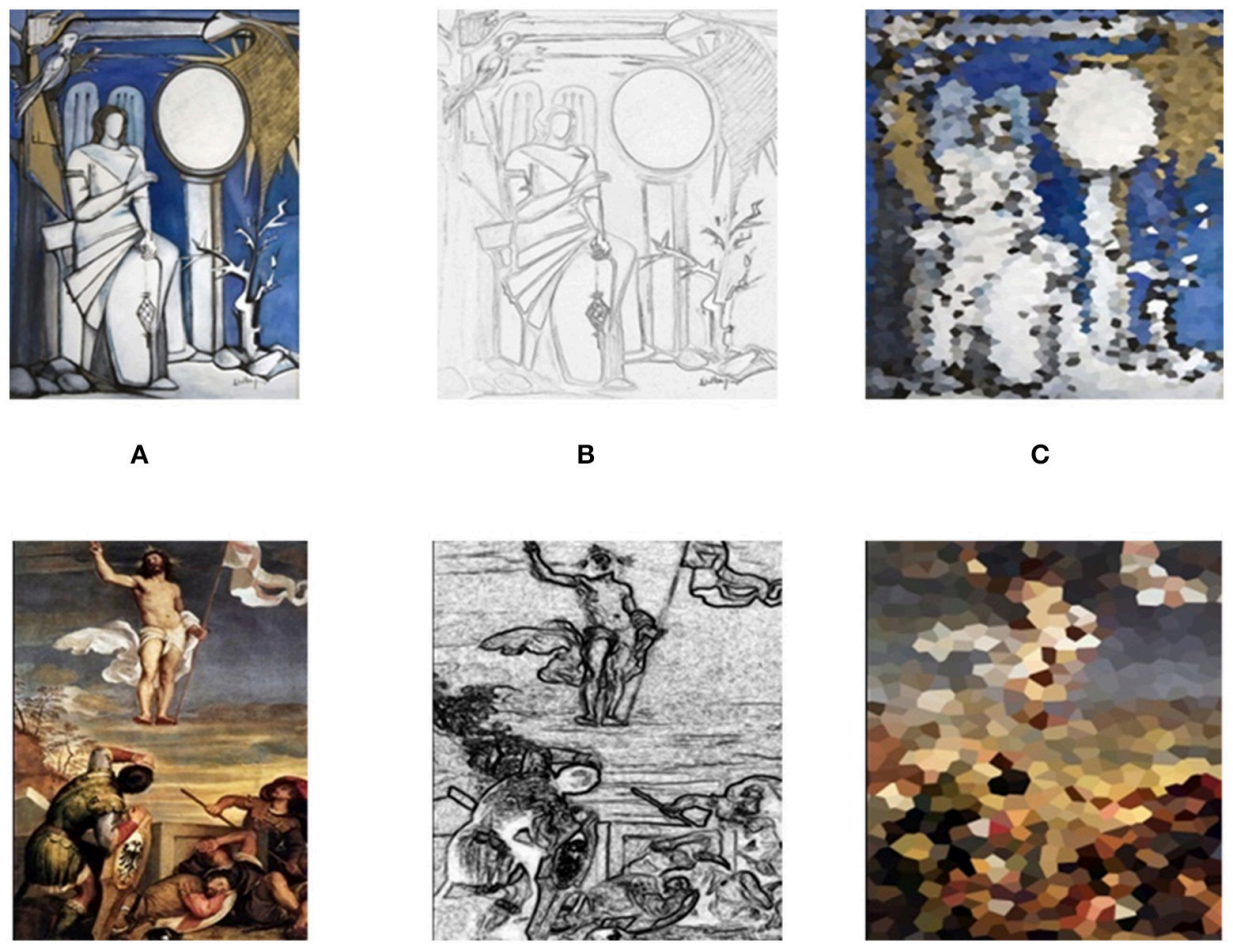
An example of the three groups of stimuli tested in the present study (Orig, column **A**; Style, column **B**; Color, column **C**). The first row presents a picture and its processed images from the contemporary artist Luisa Del Campana while the second row is related to a picture from Titian (Tiziano Vecellio) and the relative processed counterparts. Subjects were exposed to the proposed stimuli in a random order.

The first set of stimuli proposed to the participants consisted of six images of paintings taken from the works of the ancient Italian painter Titian (Tiziano-Vecellio, 1490-1576) and six images of paintings from the works of the contemporary Italian painter Luisa del Campana (1950). This set of the original pictures is named hereafter Orig. From the Orig set of pictures, other two sets were created in two separate and distinct way. In the first set, the pictures of the Orig set were processed with the use of distortion and crystallization filters to destroy all the recognizable shapes, leaving unchanged only the hues and colors employed. This set of pictures is called hereafter Color dataset. The second set of pictures were obtained from the Orig one by using the stylize effect to discard the original colors, leaving unchanged the shape of the images. Such set is hereafter referred as Style dataset. All these effects were realized on the images of the Orig original set with the use of Paint.net software. An example of the pictures included in the Orig, Color, and Style datasets is presented in Figure 1.

### Experimental setup

All the images from the three datasets (Orig, Color, Style) were presented to the subjects in a randomized order on a high definition screen for a duration of 30 s with the use of Presentation software. Subjects were seated on a comfortable chair, with a viewing distance from the screen of about 70 cm. They were instructed to stay quiet during the appreciation of the pictures, to minimize the occurrence of muscle contractions or other movement artifacts that could afflicts the neuroelectrical recordings. Immediately after each picture presentation, subjects were asked to generate an explicit judgement of the picture observed with a score ranging from 1 (very ugly) to 9 (very beautiful).

Before the observation of the images, all participants were asked to have their eye closed for 2 min and then keep their eyes open for again 2 min. Data collected during such rest conditions, were used as baseline for the successive analysis.

### Acquisition of the EEG recordings and EEG signal pre-processing

The electroencephalographic (EEG) activity was recorded by two portable 32-channels system (BrainAmp, Brainproducts GmbH, Germany). The 10–20 international system were used as guide for the placement of 37 electrodes (FP1/2, AF3/4, AF7/8, F3/4, F7/8, FC5/6, FC1/2, C3/4, CP1/2, CP5/6, T7/8, P7/8, P3/4, PO3/4, O1/2, FPz, AFz, Fz, FCz, Cz, Pz, Oz). The ear lobe channel has been used as reference. The sampling frequency was 250 Hz. Electrode impedances were kept below 5KΩ. The independent Component Analysis (ICA), and the notch filter (centered on 50 HZ) were used to remove muscular artifacts and components due to eye movements from the acquired EEG data. Visual EEG inspection was performed before the application of ICA data analysis to remove EEG segments afflicted by large artifacts. The minimum length of the artifact-free EEG recording included in the analysis was 1 min (even if not consecutive) for each participant (Babiloni et al., 2001, 2002, 2004; Cincotti et al., 2008; Borghini et al., 2014). The closed eyes condition was used to determine the Individual Alpha Frequency (IAF; Klimesch, 1999; Babiloni et al., 2000; Smith and Smith, 2001; Astolfi et al., 2008) needed to estimate for each subject the range of the EEG frequency bands of interest.

### Estimation of EEG power spectra and cortical connectivity

Processed time-series data were transformed into the frequency domain by a 256-point fast Fourier transform with Welch's method. Specifically, 30-s spans of EEG data were analyzed with a 256-point moving window without overlap. Windowed data were extended to 256 points by zero-padding to calculate power spectra, yielding an estimation of the power spectra with 60 frequency bins from 1 to 30 Hz (frequency resolution: 0.5 Hz). EEG power spectra of these windows were averaged and converted to a logarithmic scale. The following frequency bands were considered: theta (IAF-6 IAF-2) and alpha (IAF-2 IAF+2). To evaluate the cortical three-dimensional distribution of current density was used Loreta software. The method chosen was been the exact low resolution brain electromagnetic tomography (eLORETA) (Pascual-Marqui et al., 2011). In fact, eLoreta is a validated method for localizing the electric activity in the brain based on multichannel surface EEG recordings (Pascual-Marqui et al., 1994, 2011; Horacek et al., 2007; Müller et al., 2016). For all task conditions (Orig, Style, Color), we explored the coupling between brain areas within particular frequency bands based on the up-to-date coherence algorithm, named isolated effective coherence (iCoh) (Cannon et al., 2011), which is a multivariate approach to address the effective connectivity. An additional advantage of the iCoh estimates is that they are insensitive to volume conduction. A two-step procedure was applied to estimate the cortical connectivity:first, the Source Information Flow Toolbox (SIFT) (Delorme et al., 2011) in the EEGLAB was used to identify the optimal multivariate autoregressive model for the gathered EEG data. Then, the magnitude of iCoh for channel j → channel i at the frequency of w was estimated from the following formula (Cannon et al., 2011).

(1) $${\text{iCoh}}_{\text{j}\to \text{i}}\left(\text{w}\right)=\;\frac{{\left[{\text{S}}_{\text{e}}\right]}_{\text{ii}}^{-1}|\left[\hat{\text{A}}{\left(\text{w}\right)}_{\text{ij}}\right]{|}^{2}}{{\left[{\text{S}}_{\text{e}}\right]}_{\text{ii}}^{-1}|\left[\hat{\text{A}}{\left(\text{w}\right)}_{\text{ij}}\right]{|}^{2}+{\left[{\text{S}}_{\text{e}}\right]}_{\text{jj}}^{-1}|\left[\hat{\text{A}}{\left(\text{w}\right)}_{\text{jj}}\right]{|}^{2}}$$

where 0 ≤ iCoh_j → i_(w) ≤ 1, the autoregressive coefficients [A(w)]_kl_ = 0, for all (k,l) such that (k,l) ≠ (i,j) and k ≠ l and the spectral density matrix [S_εkl_] ≡ 0, for all (k,l) such that k ≠ l.

### Estimation of brain connectivity indexes from the graph theory

Application of iCoh to the gathered EEG data returns a networks of cortical connectivity depicting a fully connected graph where nodes are the electrodes and the arc are the estimated values of iCoh. The adjacency matrix A contains the information about the connectivity structure of the graph. In literature, the problem of thresholding a fully connected graph has been faced since almost a decade (De Vico Fallani et al., 2008). Here, it was adopted a threshold criteria that is based on the hypothesis that all estimated connectivity patterns had the same number of connections. To compute this “optimal” connection density, we plotted the mean efficiency values from 50 random graphs with 37 nodes, corresponding to the 37 Region of Interest (ROI), namely coincident with the scalp position of the electrodes, with an increasing connection density from 0.01 to 1, i.e., from a sparsely connected to a fully connected. We then obtained 0.178 as the “optimal” connection density, which was computed as the maximum value of the difference that separates the global efficiency from the local efficiency curve. This highest separation would increase the independence between the two indexes when measuring the global and local properties of the network (De Ridder et al., 2011). By using the threshold estimated it was possible to put the entries of the adjacency matrix A (e.g., aij) to 1 if they are greater than the estimated threshold and 0 otherwise. After this computation, the matrix A from fully connected became sparse and characterized only by binary values. After this transformation of the A matrix, it was possible to estimate a measure that describes the characteristic of connectivity of the estimated brain network: the outdegree (De Vico Fallani et al., 2008). In particular, the outdegree index of an individual node is equal to the number of links outgoing to that node, which in practice is also equal to the number of neighbors of the node. Another measure used that will be used in this work is the clustering coefficient (CC), which quantifies the value of the estimated network segregation. The clustering coefficient represent the fraction of the node's neighbors that are also neighbors of each other (Pascual-Marqui et al., 2014).

### Estimation of the emotional index by EDA and HR activities

The autonomic activities, namely the Electrodermal Activity (EDA) and the Heart Rate (HR), have been recorded with the Nexus-10 system (Mind Media, Nederlands) with a sampling rate of 32 Hz. Skin conductance was recorded by the constant voltage method (0.5 V). Ag–AgCl electrodes (8 mm diameter of active area) were attached to the palmar side of the middle phalanges of the second and third fingers of the participant's non-dominant hand by means of a velcro fastener. The company also provided disposable Ag–AgCl electrodes to acquire the HR signal. Before applying the sensors to the subjects' skin, their surface have been cleaned following procedures and suggestions previously published (Watts and Strogatz, 1998; De Vico Fallani et al., 2008). EDA and HR signals have been continuously acquired for the entire duration of the experiment and then filtered and segmented with in-house MATLAB software. For to the EDA signal, we employed a Continuous Decomposition Analysis. HR signal has been obtained using the Pan Tompkins algorithm from the gathered electrocardiographic signals (Fowles et al., 1981). In the attempt to match SCL and HR signals we referred to the circumplex model of affect plane (Pan and Tompkins, 1985; Boucsein, 2012), where the coordinates of a point in the space are defined by the HR (horizontal axis) to describe the valence and by the SCL (vertical axis) to describe the arousal phenomena (Russell and Barrett, 1999). In order to have a monodimensional variable, we describe the emotional state of a subject by defining the following Emotional Index (EI):

(2) $$EI=1-\frac{\beta }{\pi }$$

where

(3) $$\beta =\{\begin{array}{l} \frac{3}{2}\pi +\pi -\theta \text{ if GSRz}\geq 0,\text{HRz}\leq 0\\ \frac{\pi }{2}-\theta \text{ otherwise} \end{array}$$

GSRz, HRz represent the Z-score variables of GSR and HR respectively; θ, in radians, is measured as arctan (HRz, GSRz), (Vecchiato et al., 2014b). Therefore, the angle is defined in order to obtain the EI varying between [–1,1]. The interpretation of the EI implies that the higher the value the more positive the emotion experienced by the subject and vice versa.

### Statistical analysis

To estimate for the power spectra maps the false positive rate under the null hypothesis of no voxel-wise differences between three given image conditions, we implemented a randomization procedure using the t_max_ approach as previously proposed for neuroimaging data (Mauss and Robinson, 2009; Vecchiato et al., 2014a). At every iteration, two randomly selected groups, each containing one-half of the subjects under investigation, were tested at each of the 2,394 voxels. At every iteration, the largest absolute t value (from a two-tailed test) was stored in a histogram. After 5,000 iterations, for each frequency band the t value cutting off the most extreme 5% of the distribution was identified (i.e., two-tailed *p* < 0.05).

For the statistical analysis of other indices, a repeated measures Analysis of Variance (ANOVA) has been performed by using as dependent variable the indices estimated from the different experimental setup. In particular, the average values of the neurometric indexes have been estimated along the two times interval of 10 and 30 s for each paint visualizations. Different factors were considered for each of the experiments performed. For instance, for the analysis of the graph indexes outdegree and cluster coefficient the factor “HEMISPHERE” was used, with two levels (right, left) together with the factor “AREAS,” with three levels (frontal, central, parietal). For the EI index the factor “ARTIST” is used with two levels (Titian, Luisa Del Campana) and the factor “IMAGE” with three levels (Color, Style, Orig) is also used. For all the analyses, the repeated measures ANOVA was performed with the Greenhouse-Geisser correction, to protect from the spherical assumption violation, where the significance is set at the 5%. The *post-hoc* tests were performed with the use of the Duncan procedure (Arndt et al., 1996).

## Results

### Behavioral results

**Figure 2:**
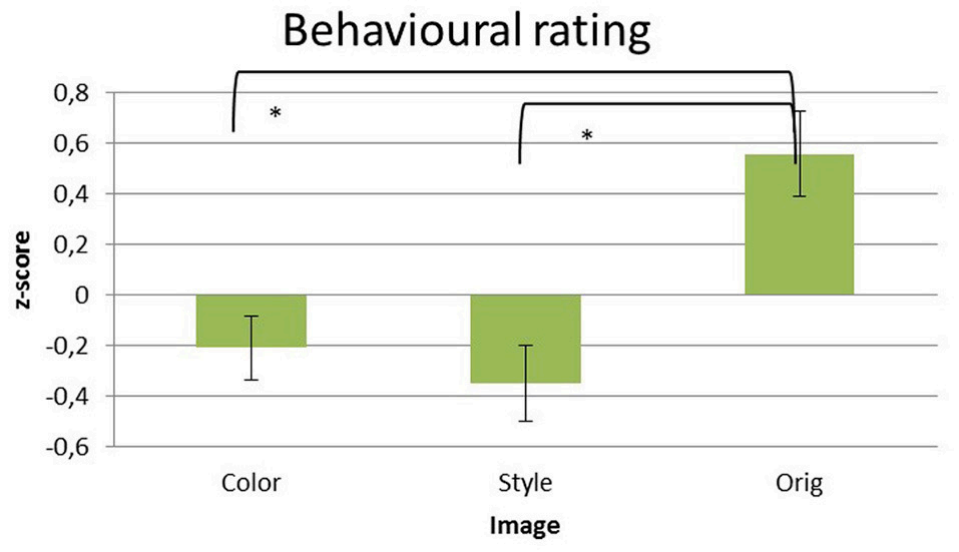
Z-score transformed average values of liking given explicitly by the subjects after the observation of each picture in the three datasets of images analyzed: Color, Style, and Orig. Error bars indicate standard deviations. Statistically significant pair-wise comparisons are highlighted with asterisks at *p* < 0.05.

Significant differences of the liking scores raised by the participants for the different pictures datasets were assessed by using a multivariate repeated measures ANOVA design with the factor IMAGE (levels: Color, Style, Orig) and the z-score of the rating values as a dependent variable. Average z-scores values of the liking scores for the pictures are reported in the Figure 2.

The statistical analysis has showed a significant difference for the factor IMAGE across the different levels [Color = (−0.209 ± 0.127), Style = (−0.35 ± 0.15), Orig = (0.558 ± 0.170); *F*_(2, 32)_ = 16.442, *p* < 0.001]. Hence, the z-score values depends of the image. Specifically, the pairwise comparisons revealed significant differences for the contrast Orig vs. Color (*p* < 0.01) and Orig vs. Styles (*p* < 0.01), whereas no difference has been found between Color and Style (*p* > 0.05). No significant differences are obtained considering the ARTIST factors (*p* > 0.05).

### Cortical patterns of power spectral density

**Figure 3:**
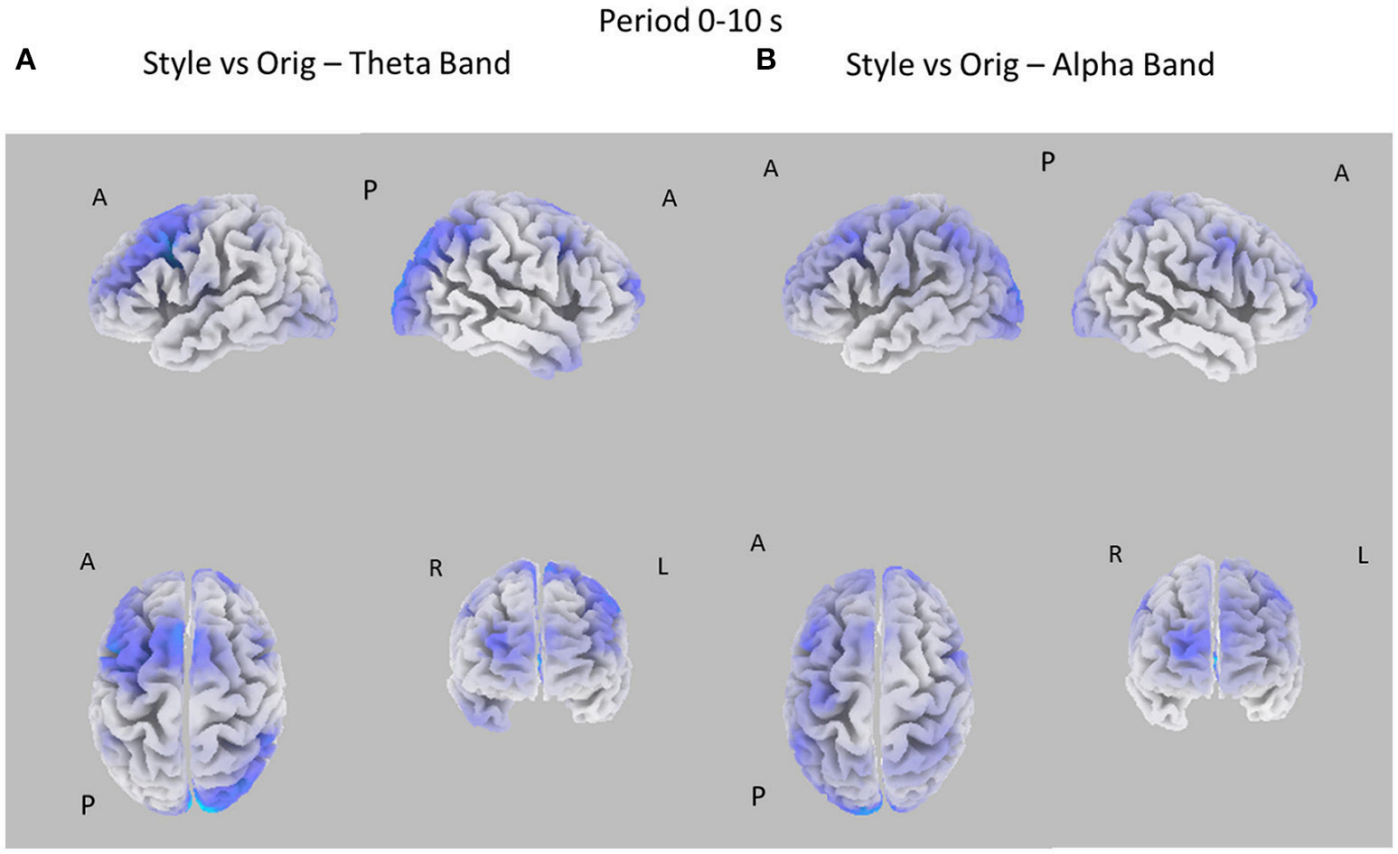
Eight cortical *t*-test maps of PSD values for the Style vs. Orig comparison in the Theta **(A)** and Alpha **(B)** band during the first 10 s of observation of the pictures. The letters close to the brain representation describe its orientation: A for anterior view, R for right side view, L for left side view, P for posterior side view. Blue color is used when the estimated cortical activity is statistically higher in the Orig than in the Style condition (*t*-values at *p* < 0.05, corrected for multiple comparisons). Gray color is used to map cortical areas where there are no significant differences between the cortical activity in the two experimental conditions.

**Figure 4:**
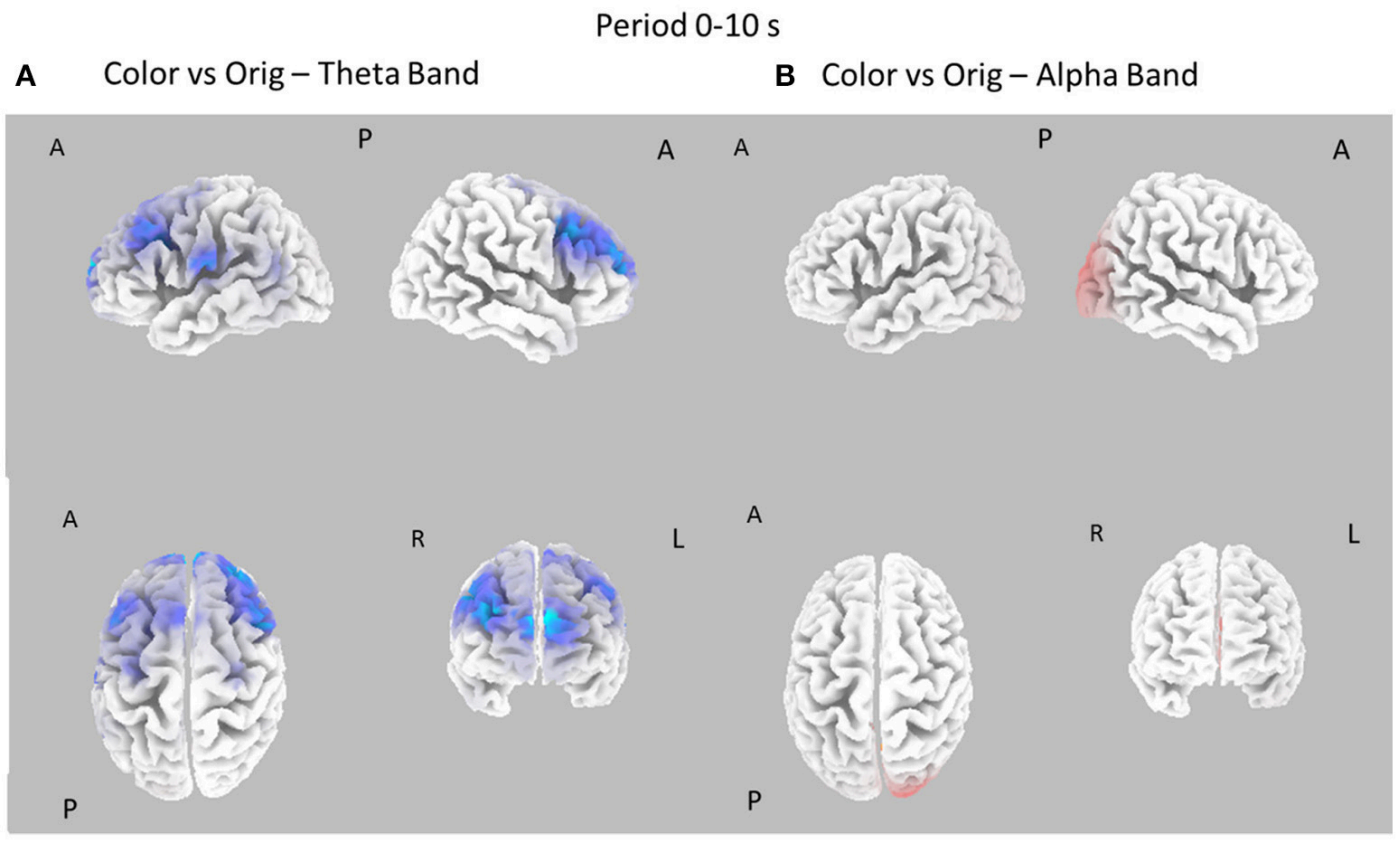
Eight cortical *t*-test maps of PSD values for the Color vs. Orig comparison in the Theta **(A)** and Alpha **(B)** band during the first 10 s of observation of the pictures. Same conventions as in Figure 3. Blue color is used when the activity is statistically higher in the Orig than in the Color condition (*t*-values at *p* < 0.05, corrected for multiple comparisons). Red color is used when the activity is statistically higher in the Color than in the Orig condition (*t*-values at *p* < 0.05, corrected for multiple comparisons).

**Figure 5:**
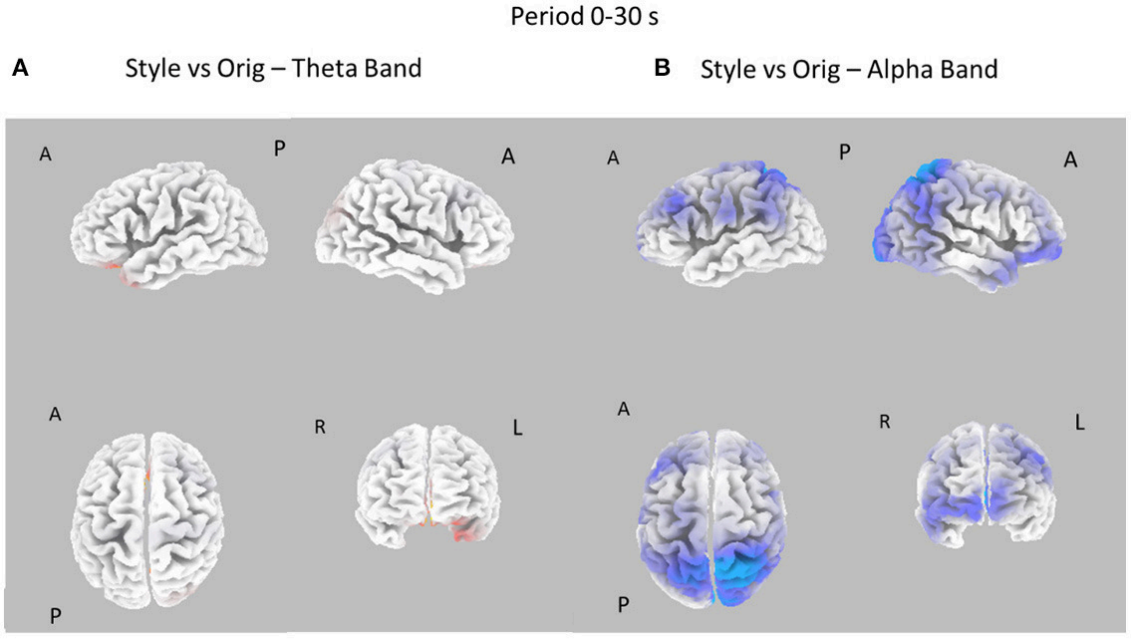
Eight cortical *t*-test maps of PSD values for the Style vs. Orig comparison in the Theta **(A)** and Alpha **(B)** band during the entire 30 s of observation of the pictures. Same conventions as in Figure 3. Blue color is used when the activity is statistically higher in the Orig than in the Style condition (*t*-values at *p* < 0.05). Red color is used when the activity is statistically higher in the Style than in the Orig condition (*t*-values at *p* < 0.05).

**Figure 6:**
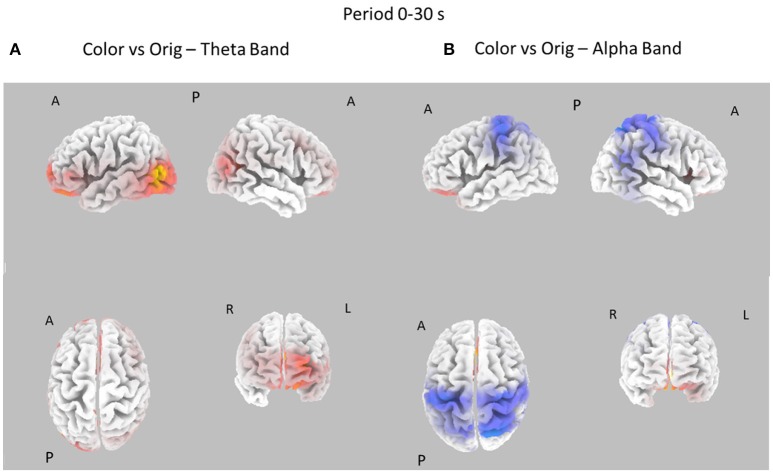
Eight cortical *t*-test maps of PSD values for the Color vs. Orig comparison in the Theta **(A)** and Alpha **(B)** band during the entire 30 s of observation of the pictures. Same conventions as in Figure 3. Blue color is used when the activity is statistically higher in the Orig than in the Color condition while red color is used when the activity is statistically higher in the Color than in the Orig condition (*t*-values at *p* < 0.05, corrected for multiple comparisons).

The EEG signals gathered during the observation of the different pictures datasets were subjected to the estimation of the cortical power spectral density by using the techniques described in the Methods section. In each subject, the cortical PSD was evaluated in the theta and alpha individual frequency bands and then contrasted between the experimental conditions. These cortical distributions of PSD were related to the two durations of the picture observation (e.g., 0–10 and 0–30 s) and for the three different images datasets (Style vs. Orig, Color, vs. Orig, Color, vs. Style). The resulting statistical spectral maps highlight cortical areas in which the estimated PSD statistically differs between the two conditions analyzed. The following Figures 3–6 present cortical maps in which the brain is viewed from different perspectives, namely frontal, back, left, and right. The maps are relative to the statistical contrast regarding the image conditions in the frequency bands of interest. The color scale on the cortex codes the statistical significance: where there are cortical areas in which the EEG power spectrum does not differ between the two conditions, the gray color is used. The red color presents statistically significant power spectral activity greater in the first condition with respect to second, while the blue color codes the opposite situation.

Figure 3 presents the eight statistical cortical maps related to the comparisons of PSD values in the Theta and Alpha bands for the Style vs. Orig condition during the first 10 s of image visualization. Specifically, it is possible to observe an increase of PSD across frontal and central areas in the Theta and Alpha bands for the Orig condition. Findings in the Alpha band return a significant desynchronization for the Orig condition in the frontal, central, and parietal cortical regions of the left hemisphere. In addition, also occipital region of the right hemisphere accounts for a de-synchronization for the Orig condition.

Figure 4 shows the contrast between the Color and Orig conditions in the Theta and Alpha bands, in the first 10 s of observation. In this case, most of the increase of cortical PSD in the Theta band is due to the Orig condition, involving frontal cortical areas. However, a significant spot of activation for the Color condition is also visible around right occipital regions in Alpha band.

Figure 5 presents the contrast between the Style and Orig condition in the Theta and Alpha bands for the whole time interval investigated (0–30s). In this case, there are no relevant differences between the cortical activity in the two conditions for Theta band, whereas a significant desynchronization of the Alpha rhythm is visible across left frontal and parietal areas for the opposite condition Orig.

Figure 6 shows the contrast between the cortical PSD estimated in the Color and Orig conditions for the Theta and Alpha bands in the whole time interval investigated (0–30s). In this case, most of the increase of cortical PSD in the Theta band is due to the Orig condition, involving the frontal and occipital cortical areas. However, a significant spot of activation for the Color condition is also visible close to the right occipital regions in Alpha band.

### Analysis of functional connectivity patterns and degree

**Figure 7:**
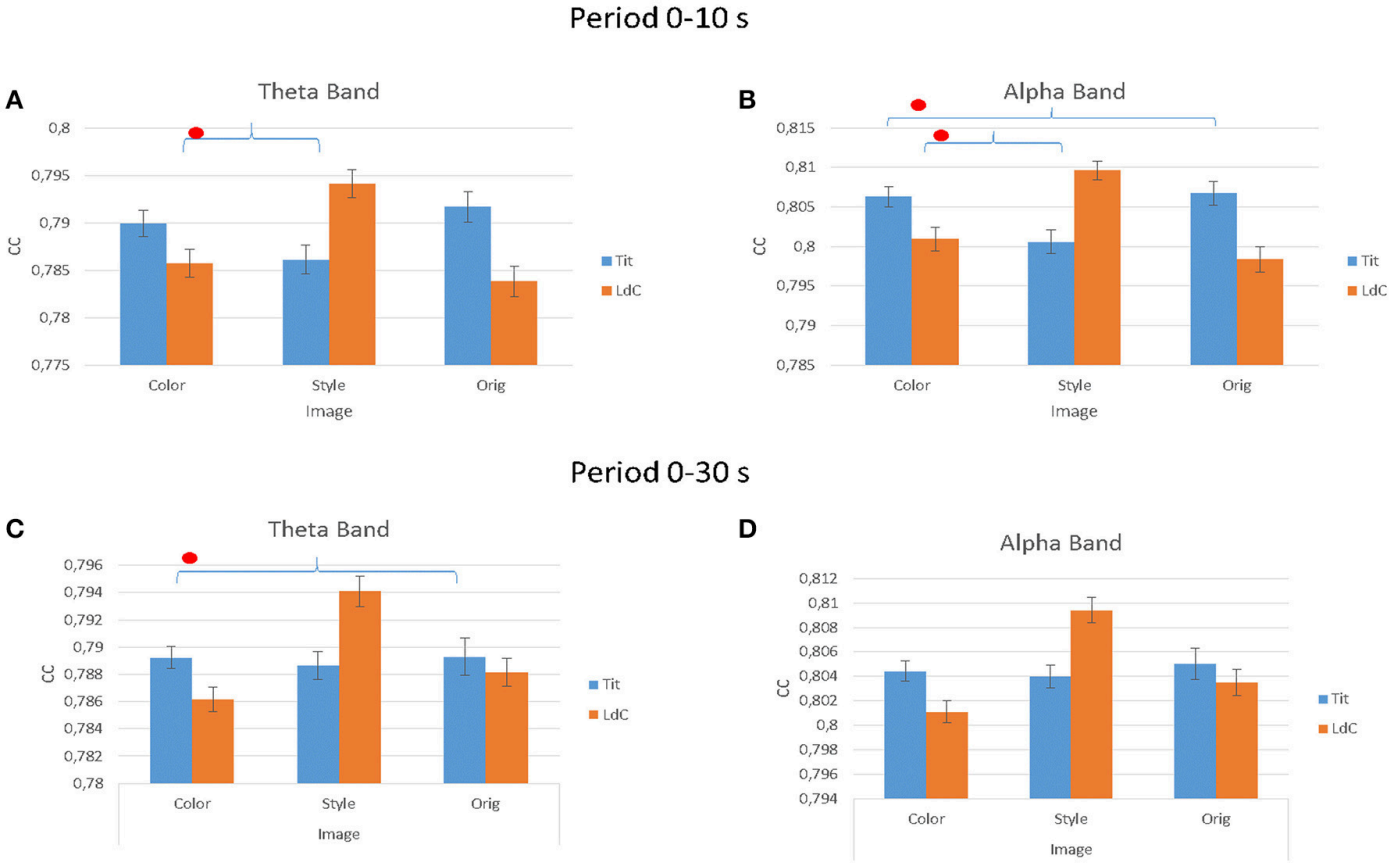
The value of the cluster coefficient (CC) estimated from the gathered EEG coherence data for different IMAGE conditions (e.g., Color, Style, Orig) and for the two Authors (e.g., Titian, Luisa del Campana). Results are provided for the first 10 s of observation **(A,B)** and for all the stimuli duration (30 s; **C,D**) in theta **(A,C)** and alpha **(B,D)** frequency bands. Stars denote a *post-hoc* statistical significance (*p* < 0.05, corrected for multiple comparisons).

The multivariate ANOVA performed on the estimated clustering coefficient index (CC) in Figure 7 indicated significant differences for factor IMAGE for all the three experimental conditions and for the two different Painters. In theta band, considering the first 10 s of painting observation, the results for the factors IMAGE are: *F*_(2, 54)_ = 106.25, *p* < 0.001; ARTIST, *F*_(1, 54)_ = 94.52, *p* < 0.001; ARTIST × IMAGE, *F*_(2, 54)_ = 1,029,54, *p* < 0.001. In the alpha band the results are IMAGE, *F*_(2, 54)_ = 125, *p* < 0.001; ARTIST, *F*_(1, 54)_ = 145, *p* < 0.001; ARTIST × IMAGE, *F*_(2, 54)_ = 1,447, *p* < 0.001. Instead during all observation experience (30 s) is been obtained: in theta band are significant the factors IMAGE, *F*_(2, 54)_ = 350.9, *p* < 0.001; ARTIST, *F*_(1, 54)_ = 9.5, *p* = 0.004; ARTIST × IMAGE, *F*_(2, 54)_ = 433.3, *p* < 0.001; ARTIST × AREAS, *F*_(3, 54)_ = 3.1, *p* = 0.036; ARTIST × IMAGE × AREAS, *F*_(6, 80)_ = 2.4, *p* = 0.033. In the alpha band are significant the following factors: IMAGE, *F*_(2, 54)_ = 619.7, *p* < 0.001; ARTIST × AREAS, *F*_(4, 56)_ = 3.2, *p* = 0.021; ARTIST × IMAGE, *F*_(2, 54)_ = 778.8, *p* < 0.001; ARTIST × IMAGE × AREAS, *F*_(8, 112_) = 2.2, *p* = 0.036.

**Figure 8:**
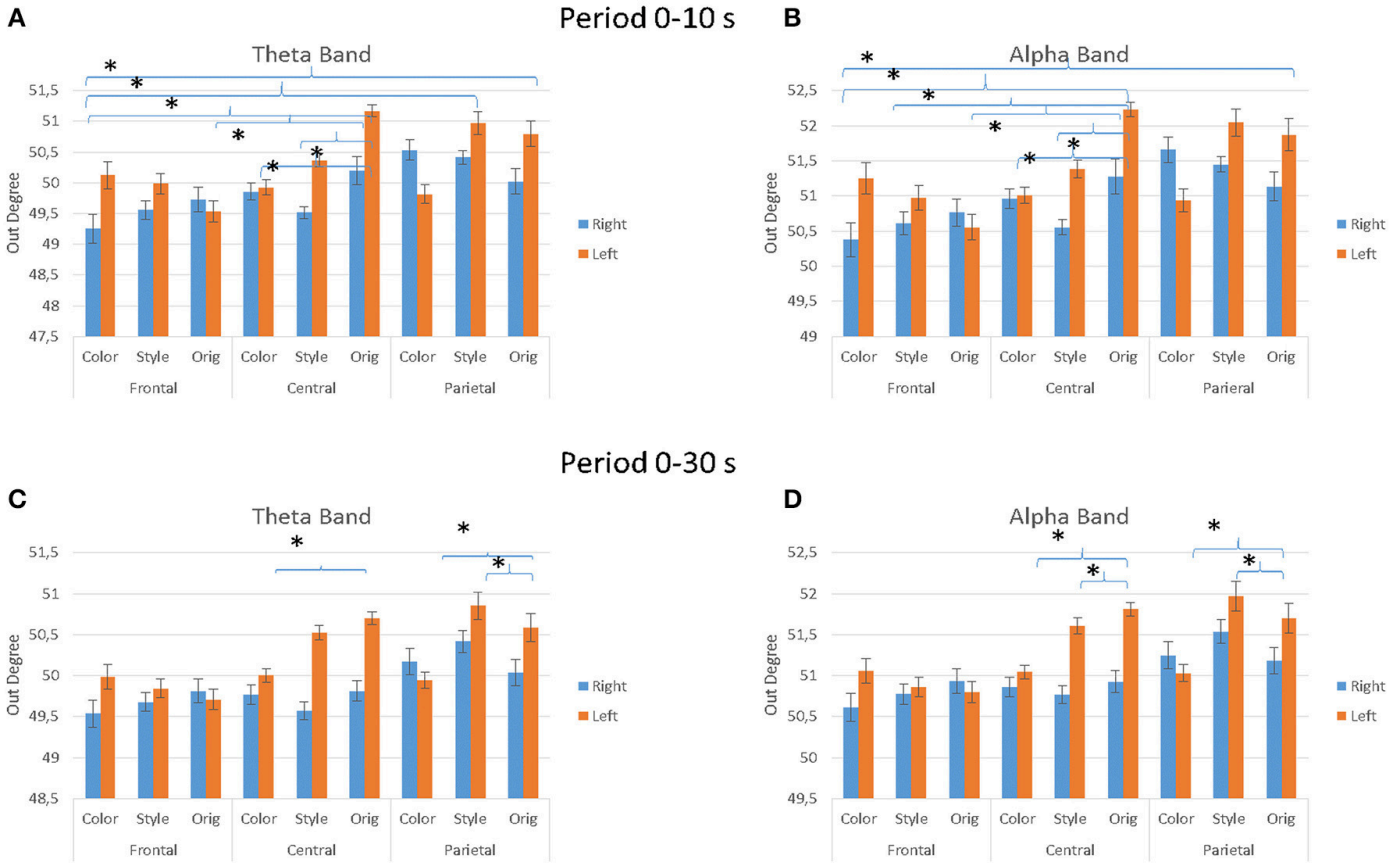
The value of the outdegree index across the different IMAGE conditions as estimated from the EEG data gathered during the first 10 s of observation **(A,B)** and for all stimuli duration (30 s; **C,D**). Results are presented for the theta frequency band **(A,C)** and for the alpha frequency band **(A,D)**. Results are also presented for different cortical zones (e.g., Frontal, Central, and Parietal) and brain hemisphere e.g., **(A,C), (B,D)**. Stars denote a *post-hoc* statistical significance (*p* < 0.05, corrected for multiple comparisons).

Figure 8 presents the average values of the outdegree index across the different factors used in the experiment (IMAGE, ARTIST, AREAS, HEMISPHERE) for the observation occurring in the first 10 s (Figures 8A,B) and for the whole duration of 30 s (Figures 8C,D). In particular, the multivariate ANOVA performed with the values of the outdegree index returns a significant differences for the factors IMAGE (Style, Color, Orig), HEMISPHERE (Figures 8A–D), ARTIST (Titian, Luisa del Campana) and AREAS (Frontal, Central, Parietal). More specifically, during the first 10 s of painting observation the results are as follows: for the theta band IMAGE × AREAS × HEMISPHERE, *F* = 2.591, *p* = 0.044. For the alpha band the results are: AREAS, *F* = 3.375, *p* = 0.015; AREAS × HEMISPHERE, *F* = 2.694, *p* = 0.04; AREAS × IMAGE, *F* = 2.365, *p* = 0.022; ARTIST × IMAGE, *F* = 5.226, *p* = 0.007; AREAS × IMAGE × HEMISPHERE, *F* = 3.523, *p* = 0.001.

During the entire duration of the observation experience (30 s) the results were for the theta band: factors IMAGE, *F* = 4.085, *p* = 0.02; IMAGE × AREAS × HEMISPHERE, *F* = 3.138, *p* = 0.019; ARTIST × IMAGE × AREAS, *F* = 4.307, *p* = 0.003. In the alpha band results are instead for the factors IMAGE, *F* = 4.677, *p* = 0.012; AREAS × IMAGE × HEMISPHERE, *F* = 2.944, *p* = 0.026; ARTIST × IMAGE × AREAS, *F* = 3.154, *p* = 0.019. Figure 8 shows also the significant differences obtained with the Duncan *post-hoc* test for each frequency band for the factors AREAS × IMAGE × HEMISPHERE.

### Analysis of emotional index

**Figure 9:**
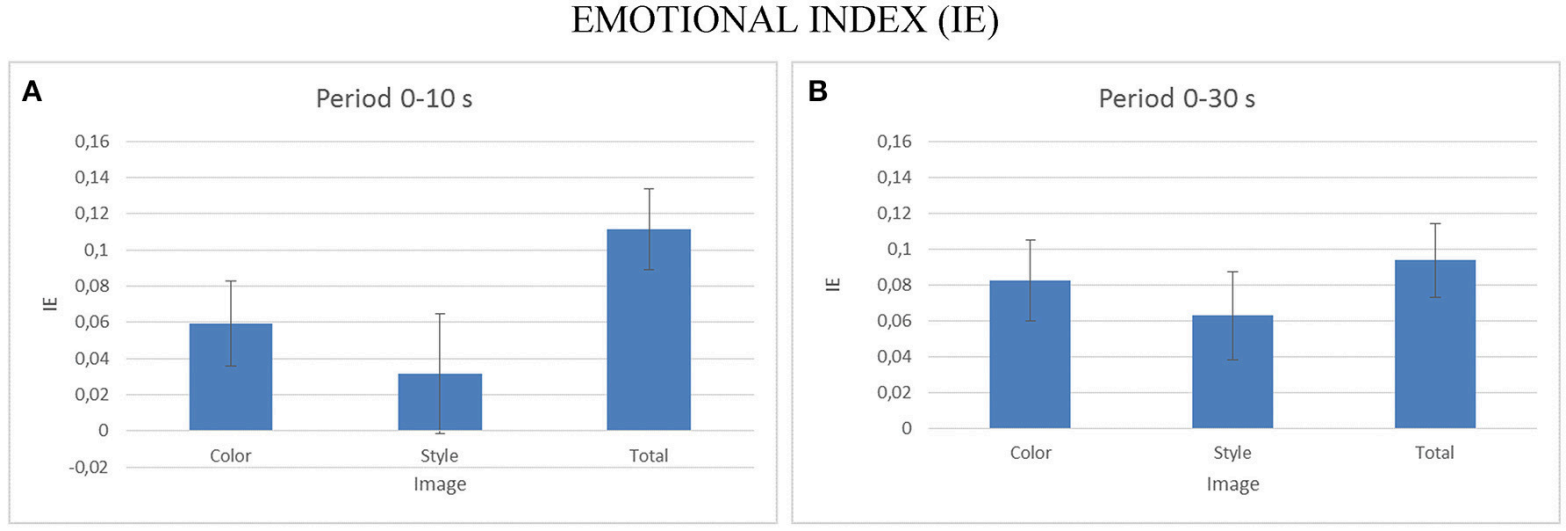
The average values of the Emotional Index (EI) estimated for all the participants across the different IMAGE dataset. Results are presented for the EI estimated during the first 10 s of observation **(A)** and for the EI estimated across all stimuli duration **(B)**. Stars denote a *post-hoc* statistical significance (*p* < 0.05, corrected for multiple comparisons).

To assess the emotional impact of the observation of the different pictures datasets, we performed a multivariate ANOVA for EI index, with the factors IMAGE (levels: Color, Style, Orig) as factor for the two duration considered (e.g., 0–10, 0–30 s). The Figure 9 presents the average values of the EI for the different levels of the IMAGE condition for the first 10 s (Figure 9B) and for all 30 s of the stimuli presentation. Statistically significant variations of the EI are obtained for the 10 s observation [*F*_(2, 18)_ = 12.25, *p* < 0.001] as well as for the 30 s one [*F*_(2, 54)_ = 6.72, *p* < 0.002]. *Post-hoc* Duncan tests revealed significant differences at *p* < 0.05 for the EI in the Orig condition for the 10 s observation and for the Style dataset for the condition of the whole duration observation.

## Discussion

### Methodologic issues

#### Limitations

The presented study has different methodological limitations we would like to discuss here. One clear limitation is related to the quality and variety of the stimuli provided. In particular, the specific figurative category chosen for the test is just one of the several existing art categories for paintings (including for instance landscapes, still-life, etc. etc.). Thus, the conclusions could be related to the specific area of paintings category described here. A second limitation is related to the enrollment of persons that had received a previous specific education in art. It may be argued in this last case that the validity of the obtained results also for normal persons has to be demonstrated. A partial mitigation for this last criticism could come from the results obtained in previous cerebral recordings of persons not trained specifically in art during the observation of figurative paintings of Jan Vermeer as well as Titian in an art gallery in Rome (Babiloni et al., 2013, 2015). In those occasions, untrained subjects in art returned equivalent level of appreciations of figurative paintings as those obtained here in the present study for the participants specifically trained in art, by using explicit scores as well as neurometric indexes. Taken together, such results suggest an equivalence of the judgment of trained and not trained persons with respect to the appreciation of figurative paintings, at least of Titian (Babiloni et al., 2015).

#### On the interpretation of EEG PSD in theta and alpha bands

Before to discuss the significance of the results obtained in this work, it could be useful to provide some remarks about the current interpretation of the cortical activity associated with the variation of EEG power in theta and alpha frequency bands (Klimesch, 1999). In particular, it has to be noted that an increase of the EEG power spectra estimated in the theta frequency band in a cortical region is associated to a parallel increase of the cortical activity in that specific cerebral area. Instead, in the case of an increase of the EEG power spectra in the alpha frequency band in a specific brain region the related cortical activity has to be considered instead decreased. In other words, in the alpha frequency bands an increase of the EEG power spectra suggests a decrement of the specific cortical activity. This was due to the “idling” nature of the EEG rhythms in such alpha band (Klimesch, 1999).

#### The unbalance of EEG PSD in alpha band on prefrontal areas and its meaning

An important aspect that will be discussed below for the interpretation of the results offered by the present study is related to the use of the EEG power spectra unbalance between left and right cortical frontal areas in the alpha band as an index of appreciation/rejection of the proposed stimuli (Davidson, 2002). In particular, previous studies have shown that the frontal cortex (FC) is anatomically and functionally connected to structures linked to the emotional processing activity in humans (Davidson and Irwin, 1999). Thus, indirect variables of emotional processing could be also gathered by tracking variations of these cerebral cortical frontal areas. In fact, although the frontal cortex is structurally and functionally heterogeneous its role in the generation of the emotions is well recognized (Davidson, 2002). EEG spectral power analyses indicate that the anterior cerebral hemispheres are differentially lateralized for approach and withdrawal motivational tendencies and emotions. Specifically, findings suggest that the left frontal and orbito-frontal cortex are an important brain area in a widespread circuit that mediates appetitive approach, while the right homologs regions appear to form a major component of a neural circuit that instantiates defensive withdrawal (Davidson, 2000, 2004). Sutton and Davidson (1997) found that greater left-sided activation predicted dispositional tendencies toward approach, whereas greater right-sided asymmetry predicted dispositional tendencies toward avoidance. In contrast, their frontal asymmetry measurement did not predict dispositional tendencies toward positive or negative emotions, suggesting an association of frontal asymmetry with approach–avoidance rather than with valence. Other sources of data converge on a similar model of frontal asymmetry. Of particular importance are studies that link anger, an unpleasant but approach-related emotion, to greater left-hemispheric activation (Harmon-Jones and Allen, 1998; Heller et al., 2002). Also, tendencies toward worry, thought to be approach-motivated in the sense of being linked to problem solving, have been linked to relatively greater left-frontal EEG activity (Harmon-Jones et al., 2006). Thus, the emerging consensus appears to be that frontal EEG asymmetry primarily reflects levels of approach motivation (left hemisphere) vs. avoidance motivation (right hemisphere), as also testified by previous studies (Usakli et al., 2009; Vecchiato et al., 2011b,a; Borghini et al., 2014).

### Comparison between the picture datasets during the first 10 s of observation

Neuroelectrical recordings suggests that for the quick responses to the observation of the Orig dataset, e.g., within the first 10 s, the level of cortical activity in the theta band is greater than those estimated for the Style and Color datasets. Increased level of theta activity have been observed in neuroelectrical recordings also during music perception judged pleasant by participants (Sammler et al., 2007). Along this line of evidence, the increased level of cortical activity in the theta frequency band here observed would be correlated with the explicit scores of the appreciation of the Orig dataset against the other two investigated. In fact, explicit scores of the participants to the study returned the appreciation of the Orig dataset when compared to the other Style and Color ones. This result is in agreement with previous findings on the appreciation of the figurative pictures when compared to other forms of art (Ramachandran and Hirstein, 1999). Along the same line of reasoning, the Orig dataset generate more Emotional activity (as estimated by the EI) than the other Style and Color datasets.

It may be argued that an increase of the cortical activity in theta frequency band could be correlated to an increased mental effort and not to the pleasantness of the stimuli perceived (Borghini et al., 2014). However, the link between the increased of EEG power in theta band and the mental effort is known to occur during the execution of complex visuo-cognitive tasks, such as in airline pilots or during difficult mathematical problem solving (Borghini et al., 2014). In the present case, it was required no particular activity to the subjects during the observation of the different picture datasets. The “pleasantness” hypothesis for the interpretation of the increase of theta activity has to be preferred then in the present context. Such hypothesis was also supported by the presence of an increase of the cortical activity in the left hemisphere in the alpha frequency band that involved the frontal areas for the Orig dataset when compared to the Style one. Such increase is consistent with the index of appreciation/rejection of the proposed stimuli in frontal areas previously discussed in Section Methodologic issues (Davidson, 2002).

The statistical significant brain activity estimated in the EEG alpha frequency band for the appreciation of the pictures dataset suggested that the cortical activity in parietal and central areas are higher for Style dataset when compared to the Orig dataset. No relevant differences in cortical activity in alpha frequency were observed for the Color and Orig dataset. Increased cortical activity in parietal areas in the alpha frequency band has been often associated to an increase of focused attention for several cognitive activity in humans (Klimesch, 1999; Borghini et al., 2014). Thus, it could be suggested that the observation of the Style dataset required more attentional effort when compared to the Orig dataset. No differences were noted between Orig and Color observation in alpha frequency band.

Graph theory results suggested that the cortical activity was spreading from the left hemisphere toward the right one, mainly from the parietal and central areas toward the frontal ones, in both theta and frequency bands. This is consistent with the generation of a judgment in the frontal areas as a results from the perception elaborated in parietal and central areas, in both the frequency domains investigated. The clustering coefficient (CC) estimated from the cortical connectivity EEG data estimated in the different dataset is a real number between zero and 1 that is zero when there is no clustering, and 1 for maximal clustering, which happens when the network consists of disjoint cliques. In this context, the clustering coefficient estimated for such condition suggest that local interconnectedness of brain networks during the initial formation of the judgment was relatively high and with high cooperative efficiency (Sporns and Honey, 2006; Bullmore and Sporns, 2009). Such pattern of clustering observed didn't change across the two investigated frequency bands.

Taken together, the values of the cerebral and autonomic data and the relative functional connectivity data suggests that during the first 10 s of the observation of the pictures:

1. The Orig dataset induced more emotion and is perceived with more appreciation than the other two Color and Style datasets;
2. The Style dataset is perceived with more attentional effort than the other investigated datasets;
3. There is evidence of a cortical flow of activity from the parietal and central areas toward the prefrontal and frontal areas during the observation of the images of all the datasets.

### Comparison between the picture datasets for the whole period (30 s) of observation

After 30 s of the observation of the pictures, the appreciation of the complete figurative paintings (Orig dataset) as estimated by the cerebral activity in theta will slightly decline and no statistical differences was noted when compared to the cerebral activity elicited by the Style dataset. An increased activity in secondary visual areas could be observed in the Color dataset when compared with the Orig one. The emotional index reports an increase of the emotions associated to the Color and Style dataset when compared to the Orig one.

In this context, the Style and the Color dataset were observed with a sustained attention when compared to the Orig dataset, as suggested by the cortical activity estimated in the alpha frequency band (Figures 4, 6). Graph theory results suggested that the cortical activity was still spreading from the left hemisphere toward the right one, mainly from the parietal and central areas toward the frontal ones, in both theta and frequency bands. It has been observed also a relative increase of the CC index across the different areas, as returned by the less number of statistical significant differences between the cortical regions involved in both frequency bands.

Taken together, the story based on the values of the cerebral and autonomic data suggests that during the entire time of observation of 30 s:

1. The emotion induced by the Color and Style datasets increased while that induced of the Orig dataset remain stable;
2. The Color and the Style dataset were perceived with more attentional effort than the Orig dataset;
3. There is evidence of a cortical flow of activity from the parietal and central areas toward the prefrontal and frontal areas during the observation of the images of all the datasets.

## Conclusion

In this study, the cortical activity correlated with the perception and appreciation of different set of pictures was estimated by using neuroelectric methodologies.

Results suggest that the verbal appreciation of the figurative pictures when compared to only their color and shape content was mainly related to the impression generated during the first 10 s of observation of the pictures. After such period of time, an increased attention effort was generated for the entire duration of the observation (e.g., up to 30 s) with the intent to further decode the pictures proposed. Such intent was perceived as rewarding since the emotions perceived by the subjects also increased (in Style and Color datasets). Such pleasure for the decoding of complex images was also suggested previously by some Authors as one of the component of the aesthetic experience (Ramachandran and Hirstein, 1999). Our result suggested an involvement of different brain areas in the appreciation of the perceived stimuli, including not only frontal areas but also motor and parietal cortices as already observed previously (Kawabata and Zeki, 2004). It must be noted that the prefrontal dorsalateral cortex (PDC) is selectively activated only by stimuli considered beautiful whereas prefrontal activity as a whole is activated during the judgment of both pleasing and unpleasing stimuli (Cela-Conde et al., 2004). The prefrontal cortex may be generally activated for directing the attention of the cognitive and perceptual mechanisms toward aesthetic perception in viewers untrained in visual arts (Ramachandran and Hirstein, 1999). In other words, related directly to a person viewing art from an aesthetic perception due to the top-down control of their cognition. The lateral prefrontal cortex is shown to be linked to higher order self-referential procession and the evaluation of internally generated information. The left lateral PFC may be involved in maintaining attention on the execution of internally generated goals associated with approaching art from an aesthetic orientation (Ramachandran and Hirstein, 1999). As previously mentioned, directing of attention toward aesthetics may have evolutionary significance. Phan et al. (2002) reported that 60% of the studies they reviewed found activation in the medial frontal cortex (mFC), whereas Murphy et al. (2003) reported the strongest localization pattern in the supracallosal ACC, both related to sadness which is a low arousal emotion. Moreover, the link between frontal midline and the activity in the anterior cingulate cortex is also shown through listening to pleasant music since ACC is activated in musical tasks (Blood et al., 1999; Blood and Zatorre, 2001). This evidence shows that ACC could be involved in processing low arousal, pleasant and withdrawal-related emotions.

On the base of results gathered in the present study, we offered the following answers to the issues raised in the Introduction section:

1. The verbal judgments given on the pictures observed have a directed relation with the cortical activity generated within the first 10 s of the observation;
2. There are differences in the neural correlates of the image perception between the two durations of 10 and 30 s relative to the images observation: in particular, the pleasantness for Style and Color datasets increased as well as the attentional effort by increasing the observation duration from 10 to 30 s. The Orig perception remain stable across the different time duration of the images observation, having the greater values of emotions and pleasantness when compared to Style and Color datasets.
3. The estimation of the cortical connectivity suggest a coherent flow of activity from parietal and motor areas toward the frontal areas. This is coherent from the notion that perception of the images with sustained visuo-motor attention (Ramachandran and Hirstein, 1999; Kawabata and Zeki, 2004) preceded the generation of the judgment about their aesthetic appreciation.

The interest of the present study is in the fact that neuroelectric imaging could be used to return useful information related to the evolution of the aesthetic judgment in humans during the perception of relative simple stimuli. It could be then used in future for the study of more complex experiments about the formation and consolidation of aesthetic judgments during the appreciation of art.

## Ethics statement

This study was carried out in accordance with the recommendations of Good Clinical Practice of Fondazione Santa Lucia with written informed consent from all subjects. All subjects gave written informed consent in accordance with the Declaration of Helsinki. The protocol was approved by the Fondazione Santa Lucia.

## Author contributions

Writing: AM and AB. Conceive and designed the experiments: FB, GV, and AT. Performed the experiment: GV and AM. Contributed materials/analysis tools: AM and DR. Analyzed the data: AM and EM.

### Conflict of interest statement

The authors declare that the research was conducted in the absence of any commercial or financial relationships that could be construed as a potential conflict of interest.
